# The prevalence and comorbidity of mental health and substance use disorders in Scandinavian prisons 2010–2019: a multi-national register study

**DOI:** 10.1186/s12888-024-05540-6

**Published:** 2024-02-05

**Authors:** Anne Bukten, Suvi Virtanen, Morten Hesse, Zheng Chang, Timo Lehmann Kvamme, Birgitte Thylstrup, Torill Tverborgvik, Ingeborg Skjærvø, Marianne R. Stavseth

**Affiliations:** 1 Department of Medical Epidemiology and Biostatistics, Karolinska Institutet, Stockholm, Sweden; 2 Norwegian Centre for Addiction Research, University of Oslo, Kirkeveien 166, Oslo, N-0407 Norway; 3 Division of Mental Health and Addiction, Oslo University Hospital, Oslo, Norway; 4 Center for Alcohol and Drug Research, Aarhus University, Aarhus, Denmark

**Keywords:** Prison, Incarceration, Mental health, Comorbidity, Record-linkage, Criminal justice, Psychiatric morbidity, Substance use disorders, Dual disorders, National registry data

## Abstract

**Background:**

Mental health disorders are common among people in prison, but their prevalence in the Scandinavian prison population remain unclear. In this multinational register study, we examined the prevalence of mental health disorders and the comorbidity of substance use disorders (SUDs) with other mental health disorders in this population. Further, we investigated how the prevalence of mental disorders at prison entry had changed in Norway, Denmark, and Sweden over the study period.

**Methods:**

The three study cohorts included all individuals, aged 19 or older, whom had been imprisoned in Norway (2010–2019), Denmark (2011–2018), and Sweden (2010–2013). Mental disorders were defined as ICD-10 diagnoses (F-codes) registered in the national patient registers. The study prevalence was estimated based on recorded diagnoses during the entire study follow-up period in each respective country. The one-year prevalence of mental disorders was estimated for each calendar year for individuals entering prison during that year.

**Results:**

The Scandinavian prison cohorts included 119 507 individuals released 191 549 times during the study period. Across all three countries a high proportion of both women (61.3%-74.4%) and men (49.6%-57.9%) had at least one mental health disorder during the observation period. The most prevalent disorders were SUDs (39.1%-44.0%), depressive disorder (8.1%-17.5%), and stress related disorder (8.8%-17.1%). Women (31.8%-41.1%) had higher levels of mental health and substance use comorbidities compared to men (20.8%-27.6%). The one-year prevalence of any mental health disorder increased over time with a 33% relative increase in Norway, 8% in Denmark, and 10% in Sweden. The proportion of individuals entering prison with a comorbid SUD and other mental disorder had also increased.

**Conclusions:**

While the incarceration rate has been decreasing during the past decade in the Scandinavian countries, an increasing proportion of people entering prison have a diagnosed mental health disorder. Our results suggest that prisons should provide adequate treatment and scale up services to accommodate the increasing proportion of people with complex health needs among incarcerated people.

**Supplementary Information:**

The online version contains supplementary material available at 10.1186/s12888-024-05540-6.

## Introduction

The prison population is increasing and has now reached around 11 million people globally [1]. Systematic reviews have shown high rates of mental health disorders in people in prison compared to the general population [2–4]. Identifying and addressing mental health disorders is a high priority for the criminal justice system and for public health in general, given that mental health disorders are associated with adverse post-release outcomes such as suicides [5], overdoses [6], and recidivism [7, 8].

Among incarcerated people, comorbidity of substance use disorders (SUDs) and other mental health disorders is common [9–11]. A recent meta-analysis estimated that nearly half of people in prison with non-affective psychosis, and more than half of those with depression had a comorbid SUD [4]. The findings imply that prevalence studies that examine one disorder at the time, which remains the norm in the literature, do not capture the full clinical picture of people who are incarcerated. Likewise, previous studies have tended to examine comorbidity in specific combinations of diagnoses (e.g., depression with SUD), and the full prevalence of comorbidity (i.e., comorbidity of any mental disorder with SUD) thus remains unclear. Comorbidity of mental health and SUDs is associated with a poorer treatment response [12], poorer adherence to medication [13], and a substantially higher risk of reoffending [8], when compared to those without comorbidities. Comorbidity poses great challenges to treatment planning, and thus it is critical that the criminal justice system has an accurate picture of the clinical health complexities of people imprisoned.

While high prevalence of mental health disorders in the prison population is a well-established finding, most prior studies have used self-report measures or interviews by a lay person to identify clinical diagnoses, which may inflate the estimates [14]. Some disorders, such as ADHD, may be particularly prone to overestimation if not diagnosed by a trained psychiatrist or psychologist [14]. In addition, retrospective studies based on interviews may be susceptible to recall bias, potentially exacerbated by stress associated with incarceration [15].

An alternative is identifying mental health problems using register-based studies. Such studies are scarce, but a register-based study reported that 63% of individuals released from Canadian prisons in 2010 had at least one psychiatric health care contact within five years preceding incarceration [16]. Further, some evidence suggests that the prevalence of psychiatric disorders in people in prison has been increasing over time [2, 4, 14, 17, 18]. However, the high heterogeneity between studies makes time trends difficult to assess in meta-analyses. If there has been an increase in mental disorders among people in prison over time, it is important to identify them in more detail, given the significant implications for the criminal justice system in terms of service development and resource allocation.

The Scandinavian countries uphold a social-democratic and humanistic perspective, asserting that individuals with mental health disorders who are deemed not accountable for their actions should not face punishment or imprisonment. In all three countries, the decision to transfer a person convicted of a crime to forensic care is ultimately made by the courts [19].

In addition, the Scandinavian countries have low imprisonment rates compared to most other countries while still having comparatively high in-prison and post-release mortality rates [20, 21]. The reason for these adverse outcomes may be that individuals who are incarcerated constitute a selected group with severe mental health problems and social vulnerabilities [22], however, no systematic investigation of the prevalence of mental health disorders in prison settings exists yet. To accommodate this, the high-quality administrative registers in the Scandinavian countries can be used to identify mental health problems in the entire prison population.

On this backdrop, the aim of this study was to use register data from Norway, Denmark, and Sweden to: 1) estimate the prevalence of clinician-diagnosed mental health disorders in the three populations, 2) estimate the yearly proportion of comorbid SUD and any other mental health diagnosis at prison entry and, 3) during the study period investigate how the prevalence of mental health disorders at prison entry has developed over time in 2010–2019.

## Methods

### National cohorts

This study is a part of the PriSUD-Nordic project [23], and included people imprisoned in Norway in 2010–2019, Denmark in 2010–2018, and Sweden in 2010–2013. The observation periods for all countries are referred to as the study period throughout this paper. Data for 2019 was not available for analysis in Denmark, and data for 2014–2019 was not available for analysis in Sweden.

The Scandinavian prison cohorts included 119 507 individuals released 191 549 times during the study period. The cohorts included people imprisoned in both high- and low security units, including people on remand (pre-trial detention). People under the age of 19, people not holding a national personal identity number (PIN), and people serving their sentence outside of prison units, e.g., community sentence and home detention with or without electronic monitoring, were excluded from the analyses.

### Prison setting

The Scandinavian countries are characterized by very low rates of imprisonment per capita. In 2021, the prison population rate per 100 000 of the national population was 57 in Norway, 72 in Denmark, and 73 in Sweden compared to 629 in the US, 165 in Australia, and 130 in the UK [24].

The Scandinavian countries have similar correctional systems: prisons are categorized into units with different levels of security, and progression through a sentence should be aimed at re-entry to the community. Moreover, prisons are publicly funded and rehabilitation-oriented, and people incarcerated are supposed to have access to universal health care and are provided opportunities for psychiatric treatment [25, 26] as well as education and rehabilitative work activities during imprisonment.

The annual Norwegian prison population is just over 3 000 (2023), including about 24% on pre-trial detention, and approximately 5% women [24]. Norway has 58 prisons classified as high and low security units and transition houses [27]. The annual Danish prison population is a little over 4 200 (2022), with about 40% on pre-trial detention, and approximately 4% women [24]. Currently, Denmark has 14 prisons classified as high- or low-security and 41 arrest houses/departments [28]. The annual Swedish prison population is just over 7 700 (2022), including 30% on pre-trial detention, and approximately 6% women [24]. Sweden has 45 prisons distributed across the country, classified as open and closed prisons.

### Data sources

The study is based on data from administrative registers in Norway, Denmark, and Sweden. Cohorts were established using prison registers from the respective countries [29]. These administrative registers contain individual-level information on each person in prison (e.g., age and sex), as well as start and end dates of each imprisonment. To estimate the prevalence of mental health disorders during the study period and prior to prison entry, cohort data from the national prison register were linked with data from the national patient register within each country. The national patient registers in all three countries are based on hospital data covering all patients receiving specialist health care, and do not cover primary care, private clinics, or treatment provided by social services or non-governmental organizations [30]. In all three countries, data were linked using unique PINs assigned to all residents.

The Norwegian Patient Register was established in 2008, and from 2009, the coverage was close to 100% [31]. The Danish National Patient Register was established in 1977 [32], and from 1995, the registry included somatic and psychiatric inpatient and outpatient care [30]. In Denmark, the Registry of Drug Abusers Receiving (DATR) (since 1996) and the National Alcohol Treatment Register (NATR) (since 2006) cover SUD treatment targeting drug use disorders and alcohol use disorders respectively [33]. The Swedish National Patient register contains all inpatient (since 1973) and outpatient (since 2001) diagnoses given from specialist public health care services nationwide.

### Mental health disorders

All mental health disorders were defined according to chapter V (F-codes) in the International Classification of Diseases and Related Health Problems, 10th Revision (ICD-10) [34]. Any mental health disorder was defined as having at least one registered F-diagnosis, F80-83, 88–89, F91-99. We categorized the specific mental health disorders according to the diagnoses in the ICD-10 classification system (e.g., organic mental disorders [F00-F09], substance use disorders [F10-F19]) (Table 1).

**Table 1:** Description of categorization for mental health disorders included in the study

Mental health disorder	ICD-10 Codes
Any mental disorder^a^	Any of the F codes specified below
Organic mental disorder	F00-F09
Substance use disorder (SUD)^b,c^	F10-F19
Schizophrenia and psychotic disorder	F20-F29
Affective disorder	
*Bipolar disorder and manic/hypomanic episode*	F30, F31, F34.0
*Depression*	F32, F33, F34 (excl. F34.0), F38, F39
Neurotic, stress-related, and somatoform disorder	
*Anxiety disorder*	F40.0, F40.1, F40.2, F41.0, F41.1
*Obsessive–compulsive disorder*	F42
*Stress-related disorder*	F43
*Other disorder*	F4 (excl. F40.0, F40.1, F40.2, F41.0, F41.1, F42, F43)
Disorders associated with physiological disturbances	F50-F59
Disorders of adult personality and behaviour	
*Dissocial personality disorder*	F60.2
*Emotionally unstable personality disorder*	F60.3
*Other disorder*	F6 (excl. F60.2, F60.3)
Intellectual disability	F70-F79
Disorders of psychological development	
*Autism spectrum disorder*	F84
Childhood onset emotional and behavioural disorder	
*ADHD*	F90
Comorbid SUD and other mental health disorder	Any SUD combined with any other specified code

^a^Not including F80-83, 88–89, F91-99

^b^Not including F17: Tobacco

^c^The Danish substance class data is based on three sources of information: data from the National Patient Register (NPR), the Drug Abusers in Treatment Register (DATR), and the National Alcohol Treatment Register (NATR). For Denmark, a SUD was coded if the person had: 1) been in hospital-based care and received an F1X diagnosis, or 2) had received treatment for a substance use disorder in either DATR or NATR

### Analyses

We examined the prevalence of several specific mental health disorders in addition to the comorbidity of SUDs and other mental health disorders. The study prevalence of diagnosed mental health disorders was defined as having any of the diagnoses registered at any time during the study period. In addition to the prevalence for each total cohort, we present estimates separately for men and women. Moreover, we estimated a one-year prevalence for each calendar year in all individuals who entered prison during that respective year. A person contributed as having a disorder if one or more diagnoses had been registered in the preceding year from first day of incarceration. For example, if a person was incarcerated on May 4^th^2012, we counted diagnoses registered between May 3^rd^2011 and May 4^th^2012. If a person was incarcerated several times during the same calendar year, any new diagnoses in the year preceding the new incarceration date were added to the calculation, but each person was counted just once for that year. To include a full year of patient data per individual during the first year of observation (2010), we retrieved patient data from the start of 2009.

To examine the changes in one-year prevalence over calendar time, we calculated the relative change (RC). The RC was calculated as a ratio; the one-year prevalence of the last year divided by the one-year prevalence of the first year. The RC can be interpreted as follows: RC < 1 indicates a decrease in one-year prevalence, RC > 1 indicates an increase in one-year prevalence, and R = 1 indicates no change.

## Results

### Cohort characteristics

The three cohorts consisted of 119 507 individuals (Norway: 50 861, Denmark: 45 532, Sweden: 23 114) and 191 549 incarcerations (Norway: 78 233, Denmark: 82 871, Sweden: 30 445). The median age at first incarceration varied between 32 to 36 years, with Denmark having the youngest average age (Table 2). Women comprised about seven percent of the Danish and Swedish populations, and a somewhat higher proportion (10.7%) in the Norwegian population. Most had one incarceration in the observation period (Norway: 71.0%, Denmark: 63.9%, Sweden: 78.7%), while a smaller portion had three or more incarcerations (Norway: 12.3%, Denmark: 18.8%, Sweden: 7.1%: note the shorter observation period for Sweden) (Table 2).

**Table 2:** Characteristics (n, %) of the prison population in Norway (2010–2019), Denmark (2010–2018), and Sweden (2010–2013)

	**Norway**		**Denmark**		**Sweden**	
Incarcerated individuals	50 861		45 532		23 114	
Number of incarcerations	78 233		82 871		30 445	
Age at first incarceration						
19—24	11 273	22.0%	13107	28.8%	4 607	19.9%
25—34	14 759	28.8%	11345	24.9%	6 421	27.8%
35—44	11 948	23.3%	9977	21.9%	4 861	21.0%
45—54	8 499	16.6%	7571	16.6%	4 570	19.8%
55—64	3 498	6.8%	2767	6.1%	2 030	8.8%
> 65	1 043	2.0%	765	1.7%	625	2.7%
Age at first incarceration^a^	34	25–45	32	24–43	36	27–48
Gender						
Female	5 429	10.7%	3370	7.4%	1 737	7.5%
Male	45 432	89.3%	42162	92.5%	21 377	92.5%
Number of incarcerations						
1	36 375	71.0%	29110	63.9%	18 193	78.7%
2	8 555	16.7%	7879	17.3%	3 272	14.2%
3	3 206	6.3%	3545	7.8%	1 121	4.9%
4	1 533	3.0%	2043	4.5%	367	1.6%
5 +	1 581	3.1%	2954	6.5%	161	0.7%
Any mental disorder	30321	59.6%	23333	51.2%	11749	50.8%

^a^Median (IQR)

### Study prevalence

The proportion of people diagnosed with any mental health disorder during the study period was high in all three countries: 59.6% in Norway, 51.2% in Denmark, and 50.8% in Sweden (Table 2). Prevalent mental health disorders were SUDs (Norway: 44.0%, Denmark: 39.9%, Sweden: 39.1%), depressive disorder (Norway: 17.5%, Denmark: 8.1%, Sweden: 10.5%), stress-related disorder (Norway: 17.1%, Denmark: 14.4%, Sweden: 8.8%), and ADHD (Norway: 11.9%, Denmark: 9.3%, Sweden: 11.8%) (Table 3).

**Table 3:** Study treatment prevalence (n, %) of mental health disorders among people imprisoned in Norway (2010–2019, *n* = 50 861), Denmark (2010–2018, *n* = 45 532), and Sweden (2010–2013, *n* = 23 114)

Mental health disorder	Norway		Denmark		Sweden	
	**n**	**%**	**n**	**%**	**n**	**%**
Organic mental disorder	1227	2.4	1031	2.3	221	1.0
Substance use disorders	22392	44.0	18177	39.9	9029	39.1
Psychosis or schizophrenia	2811	5.5	3483	7.6	821	3.6
Affective disorder						
Bipolar disorder	1559	3.1	668	1.5	424	1.8
Depressive disorder	8911	17.5	3668	8.1	2436	10.5
Neurotic, stress-related and somatoform disorder						
Anxiety disorder	4696	9.2	995	2.2	1012	4.4
Obsessive compulsive disorder	431	0.8	255	0.6	106	0.5
Stress-related disorder	8722	17.1	6576	14.4	2029	8.8
Other disorder	3680	7.2	1864	4.1	2737	11.8
Disorders associated with physiological disturbance	1006	2.0	537	1.2	397	1.7
Disorders of adult personality and behaviour						
Dissocial personality disorder	1310	2.6	1051	2.3	483	2.1
Borderline personality disorder	1373	2.7	853	1.9	282	1.2
Other disorder	3652	7.2	3027	6.6	1065	4.6
Intellectual disability	375	0.7	620	1.4	154	0.7
Disorders of psychological development						
Autism spectrum disorder	313	0.6	281	0.6	253	1.1
Childhood onset emotional and behavioural disorders						
ADHD	6028	11.9	4247	9.3	2732	11.8
Comorbid SUD and other mental health disorder	14769	29.0	10520	23.1	5024	21.7

Moreover, conditions that are infrequent in the general population, such as psychosis or schizophrenia, were also prevalent (3.6–7.6%).

The proportion of people diagnosed with comorbid SUD and other mental health disorder was 29.0% in Norway, 23.1% in Denmark, and 21.7% in Sweden (Table 3).

In all countries, the study prevalence of any mental disorder was high among women: 61.3%-74.4% of the women were diagnosed with any mental disorder, compared to 49.6%-57.9% of the men (Supplementary Table 1). Although the most frequent disorders were typically the same in men and women, the estimated prevalence of most disorders were markedly higher in women compared to men. The proportion of women diagnosed with comorbid SUD and other mental health disorder was also markedly higher among women (31.8%-41.1%) compared to men (20.8%-27.6%) (Supplementary Table 1).

### One-year prevalence

**Fig. 1:**
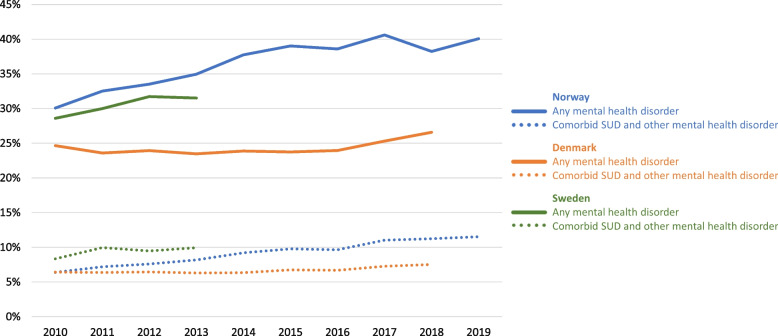
One-year prevalence of any mental health disorder and comorbidity of SUDs and other mental health disorders among people imprisoned in Norway, Denmark, and Sweden

Throughout the observation period, the average yearly prevalence estimates for any mental health disorder increased in each country: a 33% relative increase (from 30.1% to 40.1%) in Norway, an 8% relative increase (from 24.6% to 26.6%) in Denmark and a 10% relative increase in Sweden (from 28.6% to 31.5%) (Fig. 1, Table 4, Supplementary Table 2). In addition, there was an increase in comorbidity between SUDs and other mental health disorders during the study period: In Norway, there was an 80% relative increase (from 6.4% to 11.5%), in Denmark a 17% increase (from 6.4% to 7.5%), and an increase of 19% (from 8.3% to 9.9%) in Sweden (Table 4, Supplementary Table 2). In the same period, the prison population decreased in all three countries (60% change in Norway, 64% in Denmark and 87% change in Sweden) (Table 4, Supplementary Table 2).

**Table 4:**
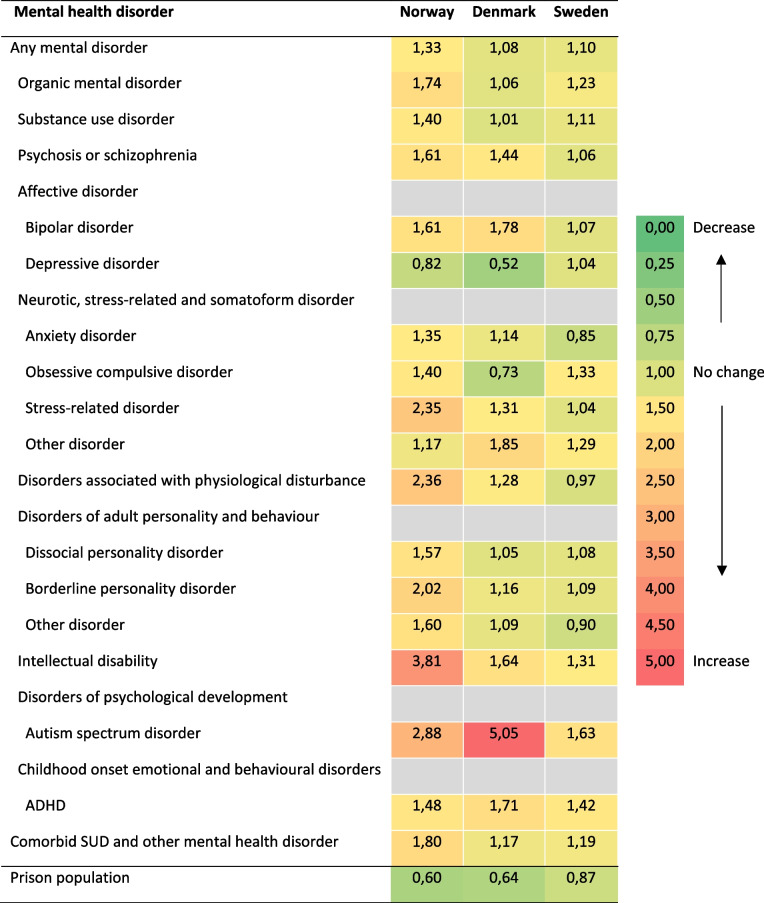
Relative change^a^ in the one-year prevalence of mental health disorders and the number of persons incarcerated per calendar year among people imprisoned in Norway (2010–2019), Denmark (2010–2018), and Sweden (2010–2013)

^a^Relative change is calculated as the one-year prevalence of the last year divided by the one-year prevalence of the first year

## Discussion

The present study utilized three large national cohorts to estimate the prevalence and comorbidity of clinician-diagnosed mental health and substance use disorders in the Scandinavian prison population. Our findings indicate that over half of the entire Scandinavian prison population received a diagnosis of at least one mental health disorder during the observation period, with a markedly higher prevalence among women. Moreover, there was an increase in the proportion of individuals entering prison with a comorbid SUD and any other mental disorder over calendar time.

During the observation period, elevated prevalence rates were identified, notably for SUDs, depressive disorders, and stress-related disorders. Additionally, there was a notable occurrence of more severe and less common conditions such as psychosis, schizophrenia, and intellectual disabilities. In the general population, schizophrenia exhibits a lifetime prevalence ranging from 0.4% to 0.7% [35], often co-occurring with SUDs [36]. A study conducted on Finnish and Swedish cohorts found that the prevalence of any SUD in patients with schizophrenia was 26% and 31%, respectively [37]. The identification of a higher prevalence among individuals with high-risk drug use, such as prison populations, therefore, aligns with expectations.

The study findings underline the need to debate current national policies regarding incarceration of individuals with severe mental disorders. The current legislations in Scandinavia considers forensic psychiatric care only in situations where people who, at the time of the criminal act, were irresponsible due to mental illness or similar conditions which affected or diminished the capability to understand the consequences and legality of their actions [19]. It could be argued that people with severe mental disorders such as schizophrenia or other psychoses do not belong in the prison system, but should rather have appropriate psychiatric care, including in forensic care if needed. Our data indeed indicate that a considerable number of individuals in this patient population ultimately face incarceration. This emphasizes the critical need to investigate alternative measures for this specific group, who are currently subjected to imprisonment.

Using a prospective study design, we found that approximately one in three people entering prison had a mental health disorder diagnosed from specialist health care services, suggesting that a high burden of mental health disorders in this population precedes prison entry. The prevalence of mental health disorders is likely to be higher, as obtaining a diagnosis in the national patient registers relies on individuals seeking help and referrals to specialist health services. Since individuals with concurrent disorders are more likely to access mental health services compared to those with substance use disorders or mental health disorders alone [38], our estimates likely represent a subset of individuals suffering from relatively severe psychopathology. Indeed, the one-year prevalence estimates in this study were significantly lower compared to other studies that employed different assessment methods [14].

In this study, we observed an increasing prevalence of diagnosed mental health disorders over time across all three countries. This finding aligns with earlier studies showing increasing prevalence in other countries, such as the US [2]. It should be noted that the absolute number of persons with mental health disorders upon prison entry remained relatively stable, while the overall number of individuals entering prison decreased. There are several plausible explanations for this pattern.

In Norway and Sweden, the decline in imprisonment rate may be attributed to a secular trend supporting alternative sanctions over imprisonment [39]. While the use of alternative sanctions has increased, individuals with mental health disorders may be less likely to receive these sanctions compared to persons without mental health disorders. In Denmark, previous studies have shown an increase in the incidence of diagnosed mental health disorders in the general population in recent years [40], suggesting that this trend may also be reflected in the prison population. However, the observed rise in incidence and prevalence of diagnosed mental health disorders in the prison population may as well reflect an increased willingness to seek treatment and/or improved treatment availability. In addition, the increase could also be attributed to easier access to screening and treatment during the specified time period.

We found that comorbid SUDs and other mental disorders were also increasing over time with an 80% relative increase in Norway, 17% in Denmark, and 19% in Sweden. A similar development has been reported in a recent meta-analysis based on studies from other countries [4]. Comorbidity poses a challenge for prison services, as coordinated care is rarely available despite being recommended as the best practice [41]. Given the increased risk of adverse outcomes in people with multiple diagnoses [8, 12, 13], early identification, appropriate treatment planning, and availability of specialized care should be considered a service development priority in prison settings [14].

We also observed a very high study prevalence of mental health morbidity. During the study period, more than 50% of the overall prison population received a diagnosis of a mental health disorder. Consistent with previous research [42], women in Scandinavian prisons appear to be a particularly vulnerable group, with up to 74% of women having at least one mental health diagnosis, up to 56% having SUDs, and 41% experiencing a comorbid diagnosis during the follow-up period. The fact that more women in prison are likely to experience SUDs is contrary to findings in the general population, where males are more likely to experience SUDs [43]. Our findings highlight how people in the prison system have complex clinical needs that are likely to persist beyond their time in incarceration, which should be considered when planning their integration into society after the sentence is served.

A strength of this study is the inclusion of three national prison populations, covering an overlapping observation period, linked to nation-wide health registers in Norway, Denmark, and Sweden, utilizing unique personal identifiers. This registry-based design covers the entire population and provides longitudinal data with controllable attrition [44, 45], minimizing the risk of selection bias and recall bias.

However, this study also has some limitations. Firstly, the prevalence rates we present cannot be directly compared to those of other studies using different methods of assessment. Since national patient register diagnoses depend on treatment seeking and referrals, our results should not be interpreted as reflecting the actual prevalence of these disorders. Secondly, the national patient registers do not capture diagnoses given in primary care or private health care services. Consequently, our data are likely to provide conservative estimates of the prevalence of diagnosed mental health disorders. Thirdly, the Swedish data covered a relatively short period, and it remains unclear whether the prevalence had developed similarly to Norway and Denmark. Lastly, as our data only covered the period from 2009 to 2019, we were unable to capture all lifetime diagnoses, i.e., those recorded before 2009.

## Conclusion

Diagnosed mental health disorders are prevalent among people in Scandinavian prisons upon entry to prison, as well as during the study period. Despite the decreasing incarceration rate in the Scandinavian countries over the past decade, a growing proportion of individuals entering prison have diagnosed mental health disorders, with many experiencing comorbidities with SUDs. Moreover, our results add to the existing literature proclaiming improved support for women's health within the prison system.

Individuals in prison should have access to the same treatment options that are available in the community [46], and our results emphasize the urgent need for access to treatment among people in prison. To improve the situation for people in prison with substance use disorders, severe mental disorders and comorbid substance use and mental disorders, the correctional services should ensure various measures both in the short and long term. Firstly, it is crucial that individuals who have received psychiatric care and SUD services before incarceration continue their treatment throughout the entire period of imprisonment. Prisons must thus provide adequate treatment and scale up services to accommodate the increasing proportion of people entering prison with complex health needs. Secondly, people should be assessed upon their arrival in prison so that treatment can be initiated for those who need it but may have been outside the treatment system before incarceration. Thirdly, correctional services must collaborate with various community agencies so that the vulnerable phase after release poses less risk to the individuals being released.

### Supplementary Information

**Additional file 1:** **Supplementary Table 1.** Study treatment prevalence (n, %) of mental health disorders among people imprisoned in Norway, Denmark, and Sweden, stratified by sex. **Supplementary Table 2.** One-year prevalence (n, %) of mental health disorders among people imprisoned in a) Norway, b) Denmark and c) Sweden.

## Data Availability

The datasets generated and/or analysed during the current study are not publicly available as the ethical approvals of this research project do not include permission to share the raw data publicly. Qualifying researchers can apply for access to relevant data in Norway, Sweden, and Denmark based on the applicable country's legislation relating to the use of register data. For any request regarding the data from this study, please contact the corresponding author.
