# Supplementary material for: The prevalence and comorbidity of mental health and substance use disorders in Scandinavian prisons 2010–2019: a multi-national register study
Source: BMC Psychiatry. 2024 Feb 5;24:95. doi: 10.1186/s12888-024-05540-6 (PMC10840271; doi:10.1186/s12888-024-05540-6)
Supplement: Supplementary file 1 — Additional file 1: Supplementary Table 1. Study treatment prevalence (n, %) of mental health disorders among people imprisoned in Norway, Denmark, and Sweden, stratified by sex. Supplementary Table 2. One-year prevalence (n, %) of mental health disorders among people imprisoned in a) Norway, b) Denmark and c) Sweden. [file 12888_2024_5540_MOESM1_ESM.docx]

Supplementary material

**Supplementary Table 1:** Study treatment prevalence (n, %) of mental health disorders among people imprisoned in Norway, Denmark, and Sweden, stratified by sex.

| **Mental health disorder** | **Norway** | | **Denmark** | | **Sweden** | |
| --- | --- | --- | --- | --- | --- | --- |
|  | **Men** | **Women** | **Men** | **Women** | **Men** | **Women** |
| Any mental disorder | 57,9 | 74,4 | 50,4 | 61,3 | 49,6 | 65,1 |
| Organic mental disorder | 2,4 | 2,8 | 2,2 | 3,4 | 0,9 | 1,1 |
| Substance use disorders | 42,6 | 55,8 | 39,7 | 43,1 | 38,0 | 51,7 |
| Psychosis or schizophrenia | 5,4 | 7,0 | 7,5 | 10,1 | 3,5 | 4,5 |
| Affective disorder |  |  |  |  |  |  |
| Bipolar disorder | 2,8 | 5,5 | 1,4 | 2,8 | 1,6 | 4,3 |
| Depressive disorder | 16,4 | 26,8 | 7,3 | 16,9 | 10,0 | 17,2 |
| Neurotic, stress-related and somatoform disorder | |  |  |  |  |  |
| Anxiety disorder | 8,7 | 14,0 | 2,0 | 4,0 | 4,2 | 6,5 |
| Obsessive compulsive disorder | 0,8 | 1,2 | 0,5 | 1,7 | 0,4 | 0,9 |
| Stress-related disorder | 15,8 | 28,8 | 13,5 | 25,8 | 8,3 | 14,5 |
| Other disorder | 6,5 | 13,2 | 3,7 | 8,7 | 11,2 | 19,3 |
| Disorders associated with physiological disturbance | 1,7 | 4,5 | 1,0 | 2,8 | 1,6 | 2,7 |
| Disorders of adult personality and behaviour | |  |  |  |  |  |
| Dissocial personality disorder | 2,7 | 1,5 | 2,4 | 1,3 | 2,2 | 1,0 |
| Borderline personality disorder | 1,9 | 9,8 | 1,2 | 10,3 | 0,9 | 5,2 |
| Other disorder | 6,8 | 10,2 | 6,1 | 13,8 | 4,4 | 6,9 |
| Intellectual disability | 0,7 | 0,9 | 1,3 | 1,8 | 0,6 | 1,2 |
| Disorders of psychological development |  |  |  |  |  |  |
| Autism spectrum disorder | 0,6 | 0,6 | 0,6 | 0,4 | 1,1 | 1,4 |
| Childhood onset emotional and behavioural disorders | |  |  |  |  |  |
| ADHD | 11,5 | 14,5 | 9,3 | 9,1 | 11,5 | 15,4 |
| Comorbid SUD and other mental health disorder | 27,6 | 41,1 | 22,4 | 31,8 | 20,8 | 33,9 |

**Supplementary Table 2:** One-year prevalence (n, %) of mental health disorders among people imprisoned in a) Norway, b) Denmark and c) Sweden

| **a) Norway** | **2010** | | | **2011** | | | **2012** | | | **2013** | | | | | **2014** | | | **2015** | | | | **2016** | | | | | **2017** | | | | | | **2018** | | | | | **2019** | | |  |
| --- | --- | --- | --- | --- | --- | --- | --- | --- | --- | --- | --- | --- | --- | --- | --- | --- | --- | --- | --- | --- | --- | --- | --- | --- | --- | --- | --- | --- | --- | --- | --- | --- | --- | --- | --- | --- | --- | --- | --- | --- | --- |
| Persons incarcerated | 9105 | | | 8456 | | | 7753 | | | 7677 | | | | | 7076 | | | 7361 | | | | 7775 | | | | | 7030 | | | | | | 6463 | | | | | 5429 | | |  |
| **Mental health disorder** | **n** | **Prev.** | **n** | | | **Prev.** | **n** | | **Prev.** | **n** | | **Prev.** | | **n** | | | **Prev.** | **n** | | **Prev.** | **n** | | | | **Prev.** | **n** | | | **Prev.** | | **n** | | | **Prev.** | | **n** | | | **Prev.** | |  |
| Any mental disorder | 2738 | 30,1 | 2749 | | | 32,5 | 2598 | | 33,5 | 2683 | | 34,9 | | 2670 | | | 37,7 | 2872 | | 39,0 | 3000 | | | | 38,6 | 2854 | | | 40,6 | | 2471 | | | 38,2 | | 2175 | | | 40,1 | |  |
| Organic mental disorder | 25 | 0,3 | 29 | | | 0,3 | 23 | | 0,3 | 30 | | 0,4 | | 27 | | | 0,4 | 32 | | 0,4 | 43 | | | | 0,6 | 45 | | | 0,6 | | 32 | | | 0,5 | | 26 | | | 0,5 | |  |
| Substance use disorder | 2147 | 23,6 | 2206 | | | 26,1 | 2119 | | 27,3 | 2210 | | 28,8 | | 2248 | | | 31,8 | 2387 | | 32,4 | 2485 | | | | 32,0 | 2319 | | | 33,0 | | 2016 | | | 31,2 | | 1789 | | | 33,0 | |  |
| Psychosis or schizophrenia | 133 | 1,5 | 113 | | | 1,3 | 122 | | 1,6 | 150 | | 2,0 | | 151 | | | 2,1 | 156 | | 2,1 | 187 | | | | 2,4 | 189 | | | 2,7 | | 156 | | | 2,4 | | 128 | | | 2,4 | |  |
| Affective disorder |  |  |  | | |  |  | |  |  | |  | |  | | |  |  | |  |  | | | |  |  | | |  | |  | | |  | |  | | |  | |  |
| Bipolar disorder | 73 | 0,8 | 91 | | | 1,1 | 75 | | 1,0 | 64 | | 0,8 | | 74 | | | 1,0 | 71 | | 1,0 | 52 | | | | 0,7 | 76 | | | 1,1 | | 63 | | | 1,0 | | 70 | | | 1,3 | |  |
| Depressive disorder | 393 | 4,3 | 356 | | | 4,2 | 321 | | 4,1 | 324 | | 4,2 | | 307 | | | 4,3 | 298 | | 4,0 | 335 | | | | 4,3 | 301 | | | 4,3 | | 262 | | | 4,1 | | 192 | | | 3,5 | |  |
| Neurotic, stress-related and somatoform disorder |  | | | |  |  | |  |  | |  | |  | | |  |  | |  |  | | |  |  | | | |  | |  | |  | | |  | |  | | |  | |
| Anxiety disorder | 154 | 1,7 | 161 | | | 1,9 | 146 | | 1,9 | 157 | | 2,0 | | 152 | | | 2,1 | 179 | | 2,4 | 195 | | | | 2,5 | 178 | | | 2,5 | | 152 | | | 2,4 | | 124 | | | 2,3 | |  |
| Obsessive compulsive disorder | 12 | 0,1 | 20 | | | 0,2 | 17 | | 0,2 | 22 | | 0,3 | | 11 | | | 0,2 | 22 | | 0,3 | 21 | | | | 0,3 | 13 | | | 0,2 | | 22 | | | 0,3 | | 10 | | | 0,2 | |  |
| Stress-related disorder | 276 | 3,0 | 282 | | | 3,3 | 253 | | 3,3 | 254 | | 3,3 | | 273 | | | 3,9 | 360 | | 4,9 | 381 | | | | 4,9 | 403 | | | 5,7 | | 397 | | | 6,1 | | 386 | | | 7,1 | |  |
| Other disorder | 120 | 1,3 | 104 | | | 1,2 | 90 | | 1,2 | 105 | | 1,4 | | 93 | | | 1,3 | 92 | | 1,2 | 105 | | | | 1,4 | 139 | | | 2,0 | | 119 | | | 1,8 | | 84 | | | 1,5 | |  |
| Disorders associated with physiological disturbance | 22 | 0,2 | 25 | | | 0,3 | 29 | | 0,4 | 30 | | 0,4 | | 17 | | | 0,2 | 34 | | 0,5 | 34 | | | | 0,4 | 37 | | | 0,5 | | 38 | | | 0,6 | | 31 | | | 0,6 | |  |
| Disorders of adult personality and behaviour |  |  |  | | |  |  | |  |  | |  | |  | | |  |  | |  |  | | | |  |  | | |  | |  | | |  | |  | | |  | |  |
| Dissocial personality disorder | 78 | 0,9 | 73 | | | 0,9 | 74 | | 1,0 | 88 | | 1,1 | | 66 | | | 0,9 | 81 | | 1,1 | 73 | | | | 0,9 | 88 | | | 1,3 | | 65 | | | 1,0 | | 73 | | | 1,3 | |  |
| Borderline personality disorder | 54 | 0,6 | 69 | | | 0,8 | 62 | | 0,8 | 55 | | 0,7 | | 49 | | | 0,7 | 69 | | 0,9 | 74 | | | | 1,0 | 80 | | | 1,1 | | 84 | | | 1,3 | | 65 | | | 1,2 | |  |
| Other disorder | 130 | 1,4 | 154 | | | 1,8 | 128 | | 1,7 | 113 | | 1,5 | | 153 | | | 2,2 | 147 | | 2,0 | 146 | | | | 1,9 | 179 | | | 2,5 | | 173 | | | 2,7 | | 124 | | | 2,3 | |  |
| Intellectual disability | 11 | 0,1 | 15 | | | 0,2 | 14 | | 0,2 | 24 | | 0,3 | | 19 | | | 0,3 | 25 | | 0,3 | 20 | | | | 0,3 | 18 | | | 0,3 | | 27 | | | 0,4 | | 25 | | | 0,5 | |  |
| Disorders of psychological development |  |  |  | | |  |  | |  |  | |  | |  | | |  |  | |  |  | | | |  |  | | |  | |  | | |  | |  | | |  | |  |
| Autism spectrum disorder | 7 | 0,1 | 9 | | | 0,1 | 12 | | 0,2 | 13 | | 0,2 | | 17 | | | 0,2 | 18 | | 0,2 | 18 | | | | 0,2 | 16 | | | 0,2 | | 17 | | | 0,3 | | 12 | | | 0,2 | |  |
| Childhood onset emotional and behavioural disorders | | | | |  |  | |  |  | |  | |  | | |  |  | |  |  | | |  |  | | | |  | |  | |  | | |  | |  | | |  | |
| ADHD | 352 | 3,9 | 324 | | | 3,8 | 314 | | 4,1 | 323 | | 4,2 | | 348 | | | 4,9 | 330 | | 4,5 | 374 | | | | 4,8 | 396 | | | 5,6 | | 335 | | | 5,2 | | 311 | | | 5,7 | |  |
| Comorbid SUD and other mental health disorder | 581 | 6,4 | 608 | | | 7,2 | 589 | | 7,6 | 627 | | 8,2 | | 651 | | | 9,2 | 719 | | 9,8 | 749 | | | | 9,6 | 775 | | | 11,0 | | 726 | | | 11,2 | | 625 | | | 11,5 | |  |

| **b) Denmark** | **2010** | | **2011** | | **2012** | | **2013** | | **2014** | | **2015** | | **2016** | | **2017** | | **2018** | |
| --- | --- | --- | --- | --- | --- | --- | --- | --- | --- | --- | --- | --- | --- | --- | --- | --- | --- | --- |
| Persons incarcerated | 10815 | | 9242 | | 9008 | | 9054 | | 8257 | | 7471 | | 6661 | | 6584 | | 6958 | |
| **Mental health disorder** | **n** | **Prev.** | **n** | **Prev.** | **n** | **Prev.** | **n** | **Prev.** | **n** | **Prev.** | **n** | **Prev.** | **n** | **Prev.** | **n** | **Prev.** | **n** | **Prev.** |
| Any mental disorder | 2665 | 24,6 | 2180 | 23,6 | 2156 | 23,9 | 2125 | 23,5 | 1971 | 23,9 | 1773 | 23,7 | 1595 | 23,9 | 1666 | 25,3 | 1849 | 26,6 |
| Organic mental disorder | 50 | 0,5 | 40 | 0,4 | 39 | 0,4 | 41 | 0,5 | 32 | 0,4 | 31 | 0,4 | 38 | 0,6 | 27 | 0,4 | 34 | 0,5 |
| Substance use disorder | 2180 | 20,2 | 1798 | 19,5 | 1751 | 19,4 | 1705 | 18,8 | 1525 | 18,5 | 1358 | 18,2 | 1227 | 18,4 | 1273 | 19,3 | 1418 | 20,4 |
| Psychosis or schizophrenia | 278 | 2,6 | 238 | 2,6 | 236 | 2,6 | 204 | 2,3 | 226 | 2,7 | 228 | 3,1 | 211 | 3,2 | 257 | 3,9 | 257 | 3,7 |
| Affective disorder |  |  |  |  |  |  |  |  |  |  |  |  |  |  |  |  |  |  |
| Bipolar disorder | 28 | 0,3 | 35 | 0,4 | 33 | 0,4 | 41 | 0,5 | 37 | 0,4 | 39 | 0,5 | 30 | 0,5 | 23 | 0,3 | 32 | 0,5 |
| Depressive disorder | 228 | 2,1 | 171 | 1,9 | 157 | 1,7 | 155 | 1,7 | 134 | 1,6 | 155 | 2,1 | 93 | 1,4 | 93 | 1,4 | 77 | 1,1 |
| Neurotic, stress-related and somatoform disorder |  |  |  |  |  |  |  |  |  |  |  |  |  |  |  |  |  |  |
| Anxiety disorder | 41 | 0,4 | 30 | 0,3 | 41 | 0,5 | 46 | 0,5 | 45 | 0,5 | 45 | 0,6 | 34 | 0,5 | 29 | 0,4 | 30 | 0,4 |
| Obsessive compulsive disorder | 15 | 0,1 | 11 | 0,1 | 13 | 0,1 | 11 | 0,1 | 16 | 0,2 | 9 | 0,1 | 6 | 0,1 | 9 | 0,1 | 7 | 0,1 |
| Stress-related disorder | 350 | 3,2 | 281 | 3,0 | 297 | 3,3 | 291 | 3,2 | 291 | 3,5 | 273 | 3,7 | 268 | 4,0 | 287 | 4,4 | 294 | 4,2 |
| Other disorder | 58 | 0,5 | 48 | 0,5 | 50 | 0,6 | 63 | 0,7 | 58 | 0,7 | 75 | 1,0 | 51 | 0,8 | 61 | 0,9 | 69 | 1,0 |
| Disorders associated with physiological disturbance | 23 | 0,2 | 27 | 0,3 | 18 | 0,2 | 17 | 0,2 | 15 | 0,2 | 9 | 0,1 | 21 | 0,3 | 21 | 0,3 | 19 | 0,3 |
| Disorders of adult personality and behaviour |  |  |  |  |  |  |  |  |  |  |  |  |  |  |  |  |  |  |
| Dissocial personality disorder | 104 | 1,0 | 66 | 0,7 | 87 | 1,0 | 86 | 0,9 | 94 | 1,1 | 69 | 0,9 | 76 | 1,1 | 63 | 1,0 | 70 | 1,0 |
| Borderline personality disorder | 51 | 0,5 | 53 | 0,6 | 51 | 0,6 | 49 | 0,5 | 52 | 0,6 | 61 | 0,8 | 36 | 0,5 | 46 | 0,7 | 38 | 0,5 |
| Other disorder | 196 | 1,8 | 163 | 1,8 | 144 | 1,6 | 154 | 1,7 | 181 | 2,2 | 137 | 1,8 | 128 | 1,9 | 134 | 2,0 | 138 | 2,0 |
| Intellectual disability | 35 | 0,3 | 19 | 0,2 | 37 | 0,4 | 22 | 0,2 | 24 | 0,3 | 41 | 0,5 | 30 | 0,5 | 38 | 0,6 | 37 | 0,5 |
| Disorders of psychological development |  |  |  |  |  |  |  |  |  |  |  |  |  |  |  |  |  |  |
| Autism spectrum disorder | 8 | 0,1 | - | - | 10 | 0,1 | 7 | 0,1 | 7 | 0,1 | 16 | 0,2 | 10 | 0,2 | 19 | 0,3 | 26 | 0,4 |
| Childhood onset emotional and behavioural disorders |  |  |  |  |  |  |  |  |  |  |  |  |  |  |  |  |  |  |
| ADHD | 246 | 2,3 | 214 | 2,3 | 234 | 2,6 | 252 | 2,8 | 263 | 3,2 | 266 | 3,6 | 222 | 3,3 | 222 | 3,4 | 270 | 3,9 |
| Comorbid SUD and other mental health disorder | 694 | 6,4 | 588 | 6,4 | 580 | 6,4 | 569 | 6,3 | 523 | 6,3 | 504 | 6,7 | 445 | 6,7 | 478 | 7,3 | 523 | 7,5 |

| **c) Sweden** | **2010** | | **2011** | | **2012** | | **2013** | |
| --- | --- | --- | --- | --- | --- | --- | --- | --- |
| Persons incarcerated | 10815 | | 9242 | | 9008 | | 9054 | |
| **Mental health disorder** | **n** | **Prev.** | **n** | **Prev.** | **n** | **Prev.** | **n** | **Prev.** |
| Any mental disorder | 2194 | 28,6 | 2192 | 30,0 | 2264 | 31,7 | 2095 | 31,5 |
| Organic mental disorder | 15 | 0,2 | 15 | 0,2 | 23 | 0,3 | 16 | 0,2 |
| Substance use disorder | 1671 | 21,8 | 1702 | 23,3 | 1731 | 24,3 | 1612 | 24,2 |
| Psychosis or schizophrenia | 94 | 1,2 | 101 | 1,4 | 92 | 1,3 | 86 | 1,3 |
| Affective disorder |  |  |  |  |  |  |  |  |
| Bipolar disorder | 52 | 0,7 | 65 | 0,9 | 62 | 0,9 | 48 | 0,7 |
| Depressive disorder | 276 | 3,6 | 261 | 3,6 | 236 | 3,3 | 249 | 3,7 |
| Neurotic, stress-related and somatoform disorder |  |  |  |  |  |  |  |  |
| Anxiety disorder | 113 | 1,5 | 90 | 1,2 | 99 | 1,4 | 83 | 1,2 |
| Obsessive compulsive disorder | 13 | 0,2 | 6 | 0,1 | 26 | 0,4 | 15 | 0,2 |
| Stress-related disorder | 247 | 3,2 | 229 | 3,1 | 251 | 3,5 | 223 | 3,4 |
| Other disorder | 301 | 3,9 | 318 | 4,4 | 320 | 4,5 | 337 | 5,1 |
| Disorders associated with physiological disturbance | 32 | 0,4 | 28 | 0,4 | 34 | 0,5 | 27 | 0,4 |
| Disorders of adult personality and behaviour |  |  |  |  |  |  |  |  |
| Dissocial personality disorder | 63 | 0,8 | 68 | 0,9 | 85 | 1,2 | 59 | 0,9 |
| Borderline personality disorder | 35 | 0,5 | 28 | 0,4 | 37 | 0,5 | 33 | 0,5 |
| Other disorder | 140 | 1,8 | 140 | 1,9 | 146 | 2,0 | 109 | 1,6 |
| Intellectual disability | 15 | 0,2 | 12 | 0,2 | 18 | 0,3 | 17 | 0,3 |
| Disorders of psychological development |  |  |  |  |  |  |  |  |
| Autism spectrum disorder | 29 | 0,4 | 34 | 0,5 | 37 | 0,5 | 41 | 0,6 |
| Childhood onset emotional and behavioural disorders |  |  |  |  |  |  |  |  |
| ADHD | 343 | 4,5 | 392 | 5,4 | 439 | 6,2 | 421 | 6,3 |
| Comorbid SUD and other mental health disorder | 640 | 8,3 | 654 | 10,0 | 676 | 9,5 | 661 | 9,9 |
